# Brazilian Savanna Fruits Contain Higher Bioactive Compounds Content and Higher Antioxidant Activity Relative to the Conventional Red Delicious Apple

**DOI:** 10.1371/journal.pone.0072826

**Published:** 2013-08-21

**Authors:** Egle Machado de Almeida Siqueira, Fernanda Ribeiro Rosa, Adriana Medeiros Fustinoni, Lívia Pimentel de Sant'Ana, Sandra Fernandes Arruda

**Affiliations:** 1 Biological Sciences Institute, Department of Cell Biology, Campus Universitário Darcy Ribeiro, Universidade de Brasília, Brasília, DF, Brazil; 2 Postgraduate Program in Human Nutrition, Faculty of Health Sciences, Campus Universitário Darcy Ribeiro, Universidade de Brasília, Brasília, DF, Brazil; 3 Nutrition Department of Health Sciences Faculty, Campus Universitário Darcy Ribeiro, Universidade de Brasília, Brasília, DF, Brazil; Nanjing Agricultural University, China

## Abstract

The bioactive compounds content and the antioxidant activity (AA) of twelve fruits native to the Cerrado were compared with the Red Delicious apple by means of the antiradical efficiency (using the 2,2-diphenyl-1-picrylhydrazil assay/DPPH), ferric reducing antioxidant power (FRAP) and the β-carotene/linoleic system. The antiradical efficiency (AE) and the kinetic parameters (Efficient concentration/EC_50_ and time needed to reach the steady state to EC_50_ concentration/TEC_50_) of the DPPH curve were also evaluated for comparison with the Trolox equivalent (TE) values. A strong, significant and positive correlation was observed between the TE and AE values, whereas a weak and negative correlation was observed between TE and EC_50,_ suggesting that the values of AE and TE are more useful for the determination of antiradical activity in fruits than the widely used EC_50_. The total phenolic content found in the fruits corresponded positively to their antioxidant activity. The high content of bioactive compounds (flavanols, anthocyanins or vitamin C) relative to the apple values found in araticum, cagaita, cajuzinho, jurubeba, lobeira, magaba and tucum corresponded to the high antioxidant activity of these fruits. Flavanols and anthocyanins may be the main bioactive components in these Cerrado fruits. The daily consumption of at least seven of the twelve Cerrado fruits studied, particularly, araticum, cagaita, lobeira and tucum, may confer protection against oxidative stress, and thus, they may prevent chronic diseases and premature aging. The findings of this study should stimulate demand, consumption and cultivation of Cerrado fruits and result in sustainable development of the region where this biome dominates.

## Introduction

Several studies have detected high antioxidant activity (AA) in fruits conventionally produced and consumed in regions of temperate climate and fertile soil, such as apples and grapes [1], [2] and this high antioxidant activity has been attributed to the high flavonoids and phenolic compounds found in these fruits [3]. However, few studies have evaluated the AA of fruits grown in an arid climate and acid soils, such as the Cerrado biome, the Brazilian savanna that houses a great diversity of plant species.

The Cerrado is distinguished by high plant diversity, with 11,046 species of phanerogams [4], of which approximately 40% are endemic [5]. The hot, semi-humid, seasonal climate with rainy summers and dry winters along with the poor, deep soil and periodic fires were probably responsible for the enormous diversity of plant and animal species in this biome. Our hypothesis is that the adverse abiotic factors, such assoil acidity, the excessive exposure to sun light and frequent fire during the dry season in addition to the constant presence of opportunistic pathogens determined the selection of species resistant to oxidative stress in the Cerrado.

Plants adapted to the adverse soil and climatic conditions of the Cerrado may have developed efficient molecular mechanisms of defense against the free radicals during their evolutionary processes. These mechanisms of defense against oxidative stress mainly take the form of bioactive compounds, especially antioxidants, and although they usually occur in small quantities in the plants, they may exert multiple actions in the human body as antioxidants, antihypertensives, anti-inflammatories or antimutagenics, among others [6], [7], [8], [9], [10].

The purpose of this study was to evaluate the bioactive compounds content and the AA of the ethyl acetate (EtOAc) and water extracts of twelve native edible portions of the Cerrado produced and consumed in the central region of Brazil in comparison to the Red Delicious apple.

Studies on the antioxidant activity of the Cerrado fruits should be encouraged, once the discovery of new sources of antioxidants can provide the development of new drugs by the pharmaceutical industry. In addition, this study may also add commercial value to the native flora of this biome, as an alternative to the sustainable development of this region and regions whose climate is similar to the Brazilian Savanna.

## Materials and Methods

### Chemicals

The following chemicals were used in this study: 2,6-dichloroindophenol (DFI), 2,2-diphenyl-1-picrylhydrazil (DPPH), 2,20-azino-bis(3-ethylbenzthiazoline-6-sulfonic acid) (ABTS), 6-hydroxy-2,5,7,8-tetramethyl chroman-2-carboxylic acid (Trolox) and 2,4,6-tris(2-pyridyl)-*S*-triazine (TPTZ). All solvents/chemicals used were of analytical grade and were obtained from Merck, Rio de Janeiro, Brazil, or Sigma-Aldrich or Sigma Chemical Co., São Paulo, Brazil.

### Fruits

The samples of the Red Delicious apple (*Malus domestica*) were obtained in the local market of Distrito Federal, Brazil from three different distributors. The samples of edible portions of twelve species native to the Cerrado (minimum of 1 kg fresh weight of each specie): araticum (*Annona crassiflora Mart*)*; baru* (*Dipteryx alata* Vog.); cagaita (*Eugenia dysenterica* DC.*);* cajuzinho (*Anacardium humile* St. Hil.); guariroba (*Syagrus oleracea* (Mart.) Becc.)*;* ingá (*Inga laurina* Willd.); jatobá (*Hymenaea stigonocarpa* Mart.); jenipapo (*Genipa americana* L.); jurubeba (*Solanum paniculatum* L.); lobeira (*Solanum grandiflorum Ruiz & Pav.*)*;* mangaba (*Hancornia speciosa* Gomes) and tucum (*Bactris setosa* Mart) were collected in the Conservation Area: Ecological Station of the University of Brasilia or in Água Limpa Farm (FAL). The national manager of this Conservation Unit belongs to the own University of Brasilia, Brazil. The permission was issued by the Instituto Brasileiro do Meio Ambiente e dos Recursos Naturais Renováveis (IBAMA)/Ministério do Meio Ambiente (Authorization N° 9/2012, IBAMA/Ministério do Meio Ambiente).

### Extraction

The extracts of the edible portions of the native species were obtained according to the method of Singh et al. (2002) [11]. The palm of guariroba and all fruits were peeled, except for apple, cagaita, cajuzinho, jurubeba and tucum. Briefly, approximately 12.5 g of the edible portions of each sample were added to 50 mL of the solvent (water or 100% ethyl acetate) and homogenized in a shaker (125 rpm) at 30°C for 1 h. The extracts were filtered under vacuum through JP41 filter paper for the removal of remaining particles and the residues were re-extracted with 50 mL of the same solvent and filtered under the same conditions. The extracts were pooled and when necessary, they were concentrated under vacuum at 40°C and stored at 70°C. The extractions were done in triplicate. The entire procedure was conducted in the dark.

### Determination of Moisture Content

Samples of the frozen fruits (12 g) were macerated in a mortar using liquid nitrogen and a porcelain pestle and gradually freeze-dried in a Liotop L101 lyophilizer, São Paulo, Brazil. The moisture content was defined as the difference between the dry weight and the wet weight and was expressed as the percentage of wet fruit.

### Determination of Bioactive Compounds

The total polyphenols in the ethyl acetate and aqueous extracts were determined according to the method of Jayaprakasha et al. (2001) [12]. The extracts were dissolved in methanol (Merck). The samples (0.2 mL) were mixed with 1.0 mL of 10-fold diluted Folin-Ciocalteu reagent and 0.8 mL of a 7.5% sodium carbonate solution. After the mixture had been allowed to stand for 30 min at room temperature, the absorbance was measured at 765 nm using a UV-visible spectrophotometer (Shimadzu, model UV-1800, Japan). The estimation of the phenolic compounds in the extracts was performed in triplicate and the results were expressed as mg of tannic acid equivalents (TAE) per 100 g of fresh fruit.

Total anthocyanins and yellow flavonoids were determined according to the method of Francis (1982) [13]. Briefly, 5 g of each fruit were extracted with 15 mL of 95% ethanol and 1.5 mol/L HCl (85:15) solution, and the absorbance was measured at 535 nm and at 374 nm for anthocyanins and yellow flavonoids, respectively. The concentration was determined using the following equations: A_535nm_ x dilution factor/98.2 and A_374nm_ x dilution factor/76.6 for anthocyanins and yellow flavonoids, respectively.

The concentration of total flavanols was determined using the *p*-dimethylaminocinnamaldehyde (DMACA) method described by Arnous et al. (2001) [14]. Briefly, 200 µL of each extract were added to 1 mL of the 0.1% DMACA in 0.1 mol/L HCl in methanol and homogenized in a vortex; after 10 min at room temperature the absorbance was measured at 640 nm. Seven-point standard curve was constructed using a catequin standard solution in a linear concentration range from 2.7 to 43.0 µg/mL. The total flavanols content was expressed as mg catequin equivalents/100 g of fresh fruit.

The vitamin C content of each fresh juice, obtained by pressing well-pulped fruit and filtering, was determined using the 2,6-dichlorophenol-indophenol (DCIP) titration method, according to the AOAC procedure [15]. The ascorbic acid concentration was calculated by comparison with the standard solution of ascorbic acid (1 mg/mL) and the results were expressed as mg ascorbic/100 g of fresh weight.

Total carotenoids were extracted and quantified using the method described by Rodrigues-Amaya (2001) [16]. About 5 g of each sample were added to 20 mL of acetone, and homogenized in a shaker (125 rpm) for 1 h. The extracts were filtered under vacuum through JP41 filter paper. Solid residue was re-extracted with 20 mL of the same solvent and filtered under the same conditions. This procedure was repeated two more times. The extracts were pooled and added to 40 mL of petroleum ether; the mixture was washed with three volumes of 300 mL of water to remove acetone. The petroleum ether phase was removed and the final volume was brought to 50 mL. The absorbance was determined at 450 nm, and the carotene quantity was calculated using the A^1%^_450 nm_  =  2,592. The results are expressed in µg β-carotene/100 g of fresh fruit.

### Measurement of antioxidant activity

#### Free Radical Scavenging Assay (DPPH)

The antiradical activity was determined according to Brand-Williams et al. (1995) [17] and modified Sánchez-Moreno et al. [18] methods, which are based on the quantification of free radical scavenging. A methanol solution containing 2.5 mg DPPH^⋅^ per 100 mL was prepared. A 100 µL aliquot of each of each extract (five different concentrations were used, ranging from 300 to 3000 ppm) was added to 2.9 mL of the DPPH^⋅^ solution. The decrease in the absorbance at 517 nm (A_517_) of different concentrations of each extract was measured in a Spectronic Genesys 2 instrument (Milton Roy) at 30 sec intervals until the DPPH absorbance stabilized. The steady-state of the DPPH^⋅^ decay reaction in the presence of the extracts was established as the point at which the slope of the decay curve was ≤ 1, i.e., (DPPH**^•^** *_t_* remaining) – (DPPH**^•^** *_t_*_-1_ remaining)/*t* − (*t* − 1) ≤ 1, where t  =  time of the A_517_ detection. The antioxidant activity was expressed as the concentration of edible portions required to reduce the original amount of DPPH^⋅^ to 50% (EC_50_) and the value was expressed as g fruit, nut or palm/kg DPPH^⋅^. The antiradical efficiency (AE) of each extract was also calculated by the equation (1/*EC_50_*.*TEC_50_*), in which TEC_50_ corresponds to the time of stabilization of EC_50_ according to Sánchez-Moreno et al. (1998) [18]. The antiradical activity was also expressed as a Trolox equivalent. The assays were conducted in triplicate and the results were expressed as the mean values ± standard deviation (SD).

#### Ferric Reducing Antioxidant Power Assay (FRAP)

The antioxidant activity of each extract was estimated via a FRAP assay according to the Benzie and Strain (1996) [19] method with modifications. Briefly, the FRAP reagent was freshly prepared by mixing solutions of 0.3 mol/L acetate buffer (pH 3.6), 10 mmol/L 2,4,6-triazine-tripyridyl in 40 mmol/L hydrochloric acid and an aqueous solution of 20 mmol/L ferric chloride in a 10∶1∶1 ratio and then incubating the mixture at 37°C for 30 min. Subsequently, 900 µL of FRAP reagent was mixed with 30 µL of fruit extract and 90 µL of deionized water. A tube containing the FRAP reagent was used as a blank solution and the absorbance at 595 nm was measured after 4 min of reaction. Aqueous solutions of known Fe (II) concentrations in the range of 100−2000 µmol/L (Fe_2_SO_4_) were used to generate a calibration curve. The total extracts were assayed in triplicate and the mean values were expressed in µmol Fe per g of fruit. The results were expressed as the mean values ± standard deviation.

#### β-Carotene/linoleic System

The antioxidant activity of each extract was estimated spectrophotometrically based on the β-carotene discoloring induced by the oxidative degradation of linoleic acid described by Jayaprakasha et al. (2001) [12] and modified by Singh et al. (2002) [11]. All of the determinations were conducted in triplicate. The decrease in the absorbance of each extract was measured at 15 min intervals for a total of 180 min The antioxidant activity (AA) of the extracts was estimated by the formula AA  =  100[1 − (*A*_0_ − *A_t_*)/(*A*°_0_ − *A*°*_t_*)], where *A*_0_ and *A*°_0_ are the absorbance values at 470 nm measured at the zero incubation time for the test sample and control, respectively and *A_t_* and *A*°*_t_* are the absorbance values measured for the test sample and control, respectively, after incubation for 180 min, according to Singh et al. (2002) [11]. The results were expressed as the % inhibition of the β-carotene oxidation reaction/g fruit, nut or palm (dry matter).

#### Statistical Analysis

The T test for independent samples was used to make comparisons between the mean values of the two extracts of each fruit and between the mean values of each fruit and of apple. The correlation between the values of the bioactive compounds and the antioxidant activity as well as between the Trolox values and each parameter of DPPH were analyzed using the Pearson test. The analysis was performed using the SPSS Statistics 17.0 program, SPSS Brazil Ltda. Significance was defined as p<0.05 and the variables are presented as the mean ± SD.

## Results and Discussion

A large number of studies have established the apple as a fruit of high antioxidant activity. Previous study showed that 100 g of fresh apples have an antioxidant activity equivalent to 1,500 mg of vitamin C, suggesting that natural antioxidants from this fruit could be more effective than a dietary supplement [20]. Sun et al. (2002) [2] investigating the profiles of total phenolics, including both soluble free and bound forms in eleven common fruits from United States of America found that with exception of cranberry, apples had the highest total phenolic content and total antioxidant activity compared to the other common fruits (for review see reference [21]). Floegel et al. (2011) [1] studying the fifty most popular antioxidant-rich foods in the United State diet identified the apple as one of the top ten foods according to their antioxidant capacities. Wolfe et al. (2008) [22] found that among fifteen fruits commonly consumed in the United States, the apples were the largest contributors of fruit phenolics in the American diet, and beside of the strawberries they were the biggest suppliers of cellular antioxidant activity.

Thus, considering the ample evidence in the literature about the antioxidant activity of apples, in the present study, the Red Delicious apple (fresh, with pods) was used as standard fruit with high antioxidant activity to compare with twelve fruits commonly consumed in the Cerrado, a brazilian savanna.

### Bioactive compounds of Fruits

#### Total Phenolic Contents of Fruit Extracts

In addition to the apples obtained in the local market, twelve samples of fruits, nut or palm native to Cerrado that grow in two states of Brazil's midwest region, Minas Gerais and Goiás and Distrito Federal, were included in this study. Some of them, such as baru, have an important economic role, both in the local market and internationally. The moisture content, expressed as a percentage of the fresh fruit and total phenolic compounds, expressed as mg TAE/100 g fruit, nut or palm (dry matter), were determined in the aqueous and EtOAc extracts of the edible portions of the samples and the results are presented in Table 1. The majority of the edible portions of the plants, including the apple, exhibited moisture contents between 70 and 93%; however, a moisture content value below 7% was found in jatobá pulp and in the baru nut.

**Table 1:** Moisture content and total phenol concentration of the ethyl acetate (EtOAc) and aqueous extracts of apple and twelve fruits native to the Brazilian Cerrado expressed as dry matter.

Common name	Species	Moisture (%)	Total phenols (mg/100 g)	Total phenols (mg/100 g)
			EtAOc	Aqueous
Apple	*Malus domestica Borkh.*	84.2	151±26	273±15^##^
Araticum	*Annona crassiflora Mart.*	70.0	580±143^*^	1,095±159^*,#^
Baru	*Dipteryx alata* Vog.	4.8	6±0^**^	53±6^***,###^
Cagaita	*Eugenia dysenterica* DC.	92.8	200±33	1203±53^***,###^
Cajuzinho	*Anacardium humile* St. Hil.	85.1	111±29	455±55^**,##^
Guariroba	*Syagrus oleracea (Mart.) Becc.*	79.5	5±1^**^	185±8^**,##^
Ingá	*Inga laurina* Willd.	74.7	34±6^**^	1,506±55^***,###^
Jatobá	*Hymenaea stigonocarpa* Mart.	6.6	5±1^**^	59±2^***,###^
Jenipapo	*Genipa americana* L.	81.1	651±61^***^	1,015±62^***,##^
Jurubeba	*Solanum paniculatum* L.	71.9	94±9^*^	1352±226^**,##^
Lobeira	*Solanum grandiflorum Ruiz & Pav.*	72.3	1,166±98^***^	344±51^###^
Mangaba	*Hancornia speciosa* Gomes	84.3	113±1	842±60^**,##^
Tucum	*Bactris setosa* Mart.	77.9	540±92^**^	3,343±664^**,##^

Means ± SD (n  =  3) followed by *, **, *** are significantly different from values obtained for apple and followed by #, ##, ### are significantly different from EtOAc extract (^*,#^ p<0.05; ^**,##^ p<0.01; ^***,###^ p< 0.001; T test, independent samples.

The levels of phenolic compounds found in the aqueous extract of apple with peel were higher than the levels found in the EtOAc extract (Table 1). These values have been reported to be within the range of 110 to 357 mg/100 g for apple extracts [2], [20], [23], [24]. The aqueous extracts of eight fruits (araticum, cagaita, cajuzinho, ingá, jenipapo, jurubeba, mangaba and tucum) and the EtOAc extracts of four fruits (araticum, jenipapo, lobeira and tucum) contained a higher level of phenolic compounds than the respective apple extracts. Notably, the total phenolic contents found in the aqueous extracts of tucum, ingá, jurubeba, cagaita and araticum were 12.2-, 5.5-, 4.9-, 4.4- and 4.0-fold higher than that of the aqueous apple extract, respectively. Additionally, the EtOAc extract of lobeira had a total phenolic content approximately 8-fold higher than that of the EtOAc apple extract (Table 1). Except for lobeira, the aqueous extracts of the other fruits studied exhibited higher total phenolic contents than the EtOAc extracts. The phenolic contents of six of the twelve studied fruits of the Cerrado (araticum, cagaita, ingá, jenipapo, jurubeba and tucum) exceed the values reported in previous studies for conventional fruits [2] and for native fruits of northeast Brazil [25].

#### Total flavanols, yellow flavonoids, anthocyanins, vitamin C and total carotenoids contents of samples extracts

The comparative analysis of the total flavanols content showed that two fruits of the Cerrado, araticum and tucum, had levels 15 to 19- fold greater than the Red Delicious apple (Table 2). With regard to the vitamin C content, samples of cagaita, cajuzinho, lobeira, mangaba, and tucum showed higher levels compared to the Red Delicious apple (Table 2).

**Table 2:** Bioactive compounds (mg/100 g of fresh matter) of apple and twelve fruits native to the Brazilian Cerrado.

			Bioactive compounds		
Fruits	Total flavanols(mg catechin/100 g fruit)	Yellow flavonoids (mg/100 g)	Total anthocyanins(mg/100 g)	Vitamin C(mg/100 g)	Total carotenoids(mg β-carotene/100 g)
Apple	37.06±2.64	5.212±0.896	2.354±0.410	26.55±3.79	0.140±0.028
Araticum	549.19±22.12^**^	4.584±0.613	0.855±0.105^**^	7.88 ±1.58^*^	0.354±0.042^**^
Baru	0.28±0.05^**^	5.056±0.296	1.974±0.157	0.22±0.00	0.100±0.009
Cagaita	2.55±0.38^***^	8.038±1.277^**^	0.468±0.047^**^	64.10±6.39^**^	0.716±0.089^***^
Cajuzinho	11.37±0.41^***^	5.597±0.889	6.905±0.453^***^	89.44±5.86^***^	0.288±0.048^*^
Guariroba	3.83±0.13^***^	4.088±0.199	0.389±0.030^**^	4.77±2.07^**^	0.021±0.005^**^
Ingá	26.18±2.38^**^	3.047±0.452^*^	0.559±0.042^**^	20.19±3.36	0.056±0.003^**^
Jatobá	9.61±0.66^***^	22.585±0.910^***^	3.441±0.400^*^	0.40±0.17	0.749±0.128^**^
Jenipapo	< LD	7.832±0.435^*^	0.219±0.050^**^	12.18±2.11^**^	0.038±0.003^**^
Jurubeba	2.56±0.26^***^	48.228±5.328^***^	4.653±0.105^**^	12.95±3.24^*^	1.362±0.153^***^
Lobeira	< LD	35.123±3.866^***^	0.195±0.019^**^	55.36±3.26^*^	0.057±0.005^**^
Mangaba	2.36±0.46^***^	6.516±0.291	0.294±0.032^**^	73.41±15.818^**^	0.359±0.010^***^
Tucum	717.56±50.67^**^	42.254±2.111^***^	83.171±6.485^**^	78.37±15.828^**^	0.148±0.020

Means ± SD (n  =  3) followed by *, **, *** are significantly different from Red Delicious apple values (^*^ p<0.05; ^**^ p<0.01; ^***^ p< 0.001; T test, independent samples. <LD: detection limit.

Five of the twelve studied fruits, cagaita, jatobá, jurubeba, lobeira and tucum showed the highest levels of yellow flavonoids, and these values were 1.5, 4.0, 9.0, 7.0 and 8.0 times higher than the value obtained for Red Delicious apple, respectively (Table 2). Although these fruits showed high levels of yellow flavanoids relative to the apple, values even higher, ranging between 65 and 200 mg/100 g of fresh matter, were found in seven tropical fruits consumed in Brazil, açaí, carnaúba, jaboticaba, jambolão, juçara, murta and puça preto [26].

Regarding to total anthocyanins content, tucum showed the highest content, 35-fold greater than the value found in the Red Delicious apple (83.17±6.48 versus 2.35±0.41 mg/100 g). High anthocyanins content was also observed in three Cerrado fruits, cajuzinho, jurubeba and jatobá compared to Red Delicious apple (Table 2). The levels of anthocyanins found in tucum were similar to the levels found in açaí, jambolão and puça-preto, which are three of the eighteen tropical fruit studied by Rufino et al. (2010) [26]. Those three fruits also have purple color as tucum.

In the present study, among all samples analyzed, the tucum seems to be the highlight, as an excellent source of bioactive compounds such as flavanols, yellow flavonoids, anthocyanins and vitamin C, compared to other samples, including the Red Delicious apple.

Considering the direct relationship between the total phenolic content and the antioxidant activity (AA) widely reported in fruits and vegetables [1], [2], the high phenolic contents found in the edible portions of Cerrado fruits suggest that they have higher AA than apple. To investigate this hypothesis we analyzed the antioxidant activity in these samples by using three methodologies.

#### Free Radical Scavenging Activity of the Extracts Using the DPPH Assay

The antiradical activity of various extracts fruits has been determined using the free radical 1,1-diphenyl-2-picrylhydrazyl (DPPH^⋅^) by the addition of various concentrations of edible portions extracts to DPPH^⋅^ (0.025 g/L to 0.415 g/L). The percentage of remaining DPPH^⋅^ was determined at 30 min or the time at which a steady state of DPPH^⋅^ was reached based on the absorbance at 515 nm Brand-Williams et al. (1995) [17]. The amount of extract required to decrease the initial DPPH^⋅^ concentration by 50% (EC_50_) has been widely used to measure the antioxidant power (free radical scavenging) of fruits [26], [27]; however, this value does not take the reaction time into account. Thus, we also determined the reaction time needed to reach the steady state at the concentration corresponding to EC_50_ along with the AE of the sample (fruits, nut or palm), a new parameter proposed by Sánchez-Moreno et al. (1998) [18] to characterize the antiradical activity of extracts. The free radical scavenging capacity was also expressed as μmol Trolox equivalent (TE)/g and the results are presented in Table 3.

**Table 3:** The free radical scavenging capacity (DPPH) in the EtOAc and aqueous extracts of the fruits expressed as the Trolox equivalent (TE), the concentration of antioxidant required to reduce the original amount of DPPH^⋅^ to 50% (EC_50_), and the antiradical efficiency (AE) estimated by the equation (1/EC_50_.TEC_50_) in which TEC_50_ corresponds to the time of stabilization of EC_50_ according to Sánchez-Moreno et al. (1998).

		EtOAc extracts				Aqueous extracts		
Commonname	Troloxμmol/g fruit	EC_50_10^3^. (g/kg DPPH)	TEC_50_min	AE1/EC_50_.TEC_50_	Trolox(μmol TE/g fruit)	EC_50_10^3^. (g/kg DPPH)	TEC_50_min	AE 1/EC_50_.TEC_50_
Apple	6.5±0.6	123.2±11.1	19.7±1.1	0.4±0.1	9.4±0.3^##^	84.7±2.9^##^	18.1±2.4	0.7±0.1^##^
Araticum	54.3±7.6^***^	14.9±2.0^**^	12.5±1.9^**^	6.0±1.5^**^	67.3±13.5^*^	12.2±2.6^***^	11.5±0.5^*^	8.2±0.8^***^
Baru	0.8±0.1^**^	1,021.8±86.8^***^	5.4±0.3^***^	0.2±0.0^*^	0.6±0.1^***,#^	1470.9±202.4^***,#^	10.5±0.8^**,###^	0.1±0.0^***,##^
Cagaita	21.5±1.4^***^	37.1±2.4^**^	9.4±0.4^***^	2.9±0.3^**^	72.7±2.0^***,###^	10.9±0.3^***,###^	6.9±0.3^*,##^	13.2±1.0^**,###^
Cajuzinho	9.3±1,3^**^	86.5±13.2^*^	13.1±0.6^**^	0.9±0.2^**^	26.5±6.6^*,#^	31.1±6.7^***,##^	7.0±0.2^*,###^	4.8±1.4^**,#^
Guariroba	< DL	< DL	< DL	< DL	< DL	< DL	< DL	< DL
Ingá	2.6±0.0^**^	303.2±0.8^**^	10.9±1.9^**^	0.3±0.1	6.2±0.7^**,#^	130.4±17.1^*,##^	24.1±1.9^*,##^	0.3±0.0^**^
Jatobá	0.2±0.1^**^	3,760.4±1299.4^**^	25.4±16.4	0.0±0.0^**^	1.7±0.0^**,##^	454.3±3.7^***,#^	9.5±3.1^*^	0.3±0.1^**,##^
Jenipapo	0.8±0.1^**^	941.8±60.4^**^	2.0±0.0^***^	0.5±0.0	0.9±0.0^***^	887.4±0.6^***^	1.9±0.1^**^	0.6±0.0
Jurubeba	1.8±0.0^**^	449.5±1.0^***^	2.0±0.0^***^	1.1±0.0^**^	26.8±0.5^***,###^	29.7±0.5^***,###^	12.9±0.5^###^	2.6±0.1^***,##^
Lobeira	66.8±14.0^**^	12.3±2.6^***^	5.1±0.3^***^	16.3±2.5^***^	7.9±3.9^##^	115.2±44.1^***^	9.2±1.9^**,#^	1.2±0.2^*,##^
Mangaba	4.2±0.0^*^	187.3±1.7^***^	12.8±0.8^**^	0.4±0.0	8.2±0.5 ^*,##^	97.3±6.6^*,###^	15.9±3.2	0.7±0.1^#^
Tucum	50.6±2.6^***^	15.8±0.8^***^	18.6±6.3	4.3±1.1^**^	75.3±10.9^***,#^	10.8±1.6^***,##^	22.1±3.3	4.6±0.2^***^

Means ± SD (n  =  3) followed by *, **, *** are significantly different from values obtained for apple and followed by #, ##, ### are significantly different from EtOAc extract (^*,#^ p<0.05; ^**,##^ p<0.01; ^***,###^ p<0.001; T test, independent samples.

According to the classification of Sánchez-Moreno et al. (1998) [18], the kinetic behaviors of the reactions observed for both aqueous and EtOAc extracts of all studied edible portions, including apple, were intermediate, as the TEC_50_ values for all reactions ranged between 1.9 to 25.4 min (data not shown). Except for lobeira and baru nut, the aqueous extracts of most species exhibited higher antiradical activity values than the corresponding EtOAc extracts as measured by Trolox equivalent (TE) or by antiradical efficiency (AE) and lower values of EC_50_ (Table 3), following the same trend observed for the concentration of the phenolic compounds.

To identify the relationship between the three kinetic DPPH parameters (EC_50_, TEC_50_ and AE, obtained by the kinetics of the reactions containing the fruit extract and DPPH) and the antiradical activity expressed as the Trolox equivalent (TE), a comparative analysis was performed using a Pearson correlation test. A strong and significant positive correlation was found between TE and AE in both the aqueous (r = 0.869 and p = 0.000) and EtOAc (r = 0.885 and p<0.000) extracts of the fruits, while a weak negative correlation was found between TE and EC_50_ (r  =  −0.436, p = 0.006 and −0.345, 0.031, respectively). No correlation was found between TE and TEC_50_.

Five EtOAc extracts and five aqueous extracts of the Cerrado fruits showed higher antiradical activity than the respective apple extracts as determined by the TE and AE parameters. Surprisingly, the TE and AE values of in the lobeira EtOAc extract were approximately 10- and 40-fold higher than those of the EtOAc apple extract, respectively. The antiradical activity measured by these two parameters (TE and AE) in the EtOAc extracts of araticum, tucum and cagaita also exceeded (approximately 8- and 15-, 7- and 10-, 3- and 7-fold higher, respectively) the corresponding values in the EtOAc extract of apple. Additionally, the antiradical activities as determined by the TE and AE parameters in the aqueous extracts of araticum, cagaita and tucum were 7- and 11-, 7- and 18-, 8- and 6-fold greater than the TE and AE of the aqueous extract of apple, respectively. The aqueous and EtOAc extracts of araticum, cagaita, cajuzinho, lobeira and tucum had EC_50_ values 3.5-fold to 40-fold lower than other studied nontraditional tropical fruits from Brazil [26], demonstrating their high antioxidant activity.

The comparative analysis between the total phenolic content and the DPPH parameters showed a strong positive correlation between the total phenolic content and the values of TE and AE in the EtOAc extracts (r = 0.804, p = 0.000 and r = 0.858, p = 0.000, respectively). In the aqueous extracts, both correlations were weak (r = 0.668, p = 0.000 and r = 0.339, p =  0.035, respectively), but also significant. No correlation was found between total phenolic content and EC_50_. These results suggest that the TE and AE values are more meaningful than the EC_50_ and TEC_50_ parameters in determining the antiradical activities of samples. These results demonstrated that high activity levels against free radicals were present mainly in the samples that contained higher levels of phenolic compounds, suggesting that these phytochemicals may be responsible for this high antiradical activity. The significant high correlation found between the levels of phenolic compounds and these two DPPH parameters reinforces the involvement of the phenolic compounds in the antioxidant activities of these fruits.

A strong and positive correlation between total flavanols content and the antioxidant activity measured by DPPH assay was observed in both aqueous (r = 0.727, p = 0.007) and EtOAc (r = 0.632, p = 0.028) extracts, suggesting that the flavanols could be the main phenolic compound responsible by the antioxidant activity of these studied fruits.

#### Ferric Reducing Antioxidant Power (FRAP)

The aqueous extracts studied, including apple, exhibited higher FRAP values than the EtOAc extracts. The EtOAc extract of tucum exhibited the highest value in the FRAP assay, it was approximately 6-fold higher than the values found in the EtOAc apple extract. The FRAP values of the EtOAc extracts of araticum, cagaita, cajuzinho and lobeira were approximately 2-fold higher than that of the apple EtOAc extract. The aqueous extracts of tucum and araticum showed the highest FRAP values (approximately 6-fold higher than the apple extract), followed by cagaita, cajuzinho, jurubeba and mangaba (Table 4). The antioxidant activities estimated by FRAP assay in aqueous extracts of araticum, cagaita, cajuzinho, jurubeba, lobeira, mangaba and tucum (39.7 to 220 µmol/g fruit) were higher than conventional fruits commonly consumed by population such as avocado, banana, grape, orange, papaya, pineapple and watermelon whose values ranged between 2.76 to 14.5 µmol/g fruit [28].

**Table 4:** Ferric reducing antioxidant power (FRAP) and inhibition of β-carotene/linoleic oxidation assays.

Common name	FRAP	(µmol/g of fruit)	β-Carotene/linoleic	(% Inhibition/g)
	EtOAc	Aqueous	EtOAc	Aqueous
Apple	8.6±1.7	33.8±3.9^##^	4.0±0.3	3.2±0.6
Araticum	16.0±1.7^**^	212.0±27.5^***,##^	2.3±0.1^**^	17.0±1.5^***,##^
Baru	1.3±0.1^*^	8.8±0.2^***,###^	0.6±0.1^***^	6.0±0.5^**,##^
Cagaita	19.6±1.0^**^	107.0±2.0^***,###^	0.6±0.4^***^	16.4±2.0^***,###^
Cajuzinho	18.5±0.5^**^	45.5±1.9^*,###^	1.9±0.4^**^	0.7±0.3^**,#^
Guariroba	2.2±0.8^**^	8.8±1.6^**,##^	1.5±0.4^**^	0.7±0.1^**,#^
Ingá	2.0±0.3^*^	14.9±3.3^**,##^	0.1±0.1^***^	0.4±0.2^**,#^
Jatobá	0.7±0.1^*^	5.4±1.3^***,##^	0.4±0.0^***^	0.4±0.1^**^
Jenipapo	1.3±0.2^*^	8.2±3.0^**,#^	1.1±0.1^***^	0.4±0.2^**,##^
Jurubeba	8.6±0.3	54.1±0.4^**,###^	0.7±0.3^***^	4.7±0.2^*,###^
Lobeira	17.5±0.7^**^	39.7±0.6^###^	−0.3±0.5^**^	45.1±1.2^***,###^
Mangaba	7.4±2.2	47.2±2.3^**,###^	5.7±0.1^**^	5.3±0.9^*^
Tucum	53.2±3.9^***^	220.0 ±19.6^***,###^	6.0±0.5^**^	19.9±3.0^**,#^

Means ± SD (n  =  3) followed by *, **, *** are significantly different from apple value; and followed by #, ##, ### are significantly different from EtOAc extract (^*,#^ p<0.05; ^**,##^ p<0.01; ^***, ###^ p< 0.001; T test, independent samples).

In this study, we observed a high and significant correlation between the levels of total phenolic content and the FRAP values in the aqueous extracts of the fruits (r = 0.689 and p = 0.000). In the EtOAc extracts, this correlation was lower, but it was still significant (r = 0.404 and p = 0.011). A strong and positive correlation between total flavanols content and the antioxidant activity measured by FRAP assay was observed in both aqueous (r = 0.918, p = 0.000) and EtOAc (r = 0.765, p = 0.004) extracts of the fruits. Anthocyanins levels also positively correlated with FRAP values in aqueous (r = 0.630 p = 0.028) and in EtOAc extracts (r = 0.873, p = 0.000) of the fruits. These results reinforce that flavanols could be the main antioxidant compounds, but the anthocyanins may also contribute to antioxidant activity of these fruits.

#### Antioxidant Activity of Fruits Using the β-Carotene/linoleic Acid System

The antioxidant activity was also evaluated by the β-Carotene/linoleic assay, and the results, expressed as the percent inhibition of β-carotene oxidation, are shown in Table 4. Following the trend observed in the DPPH and FRAP assays, the aqueous extracts of most species studied inhibited the oxidation of β-carotene to a greater extent than the EtOAc extracts. Notably, the percentage of inhibition induced by the aqueous extract of lobeira was approximately 14-fold higher than that of the apple aqueous extract. The aqueous extract of tucum had the second highest value of antioxidant activity in this assay, approximately 6-fold higher than the aqueous apple extract, and was followed by araticum and cagaita, which inhibited β-carotene oxidation approximately 5-fold more extensively than the apple aqueous extract. The aqueous extracts of baru nut, mangaba and jurubeba also exhibited higher antioxidant activities in this assay than apple (p<0.05). Except for tucum and mangaba, the EtOAc extracts from the other fruits showed antioxidant activities equal to or lower than the apple EtOAc extract (Table 4). No correlation was observed between the levels of total phenolic content and the antioxidant activity measured in the β-Carotene/linoleic assay (% inhibition) in both the EtOAc and aqueous extracts. No correlation was obtained between β-Carotene/linoleic assay (% inhibition) and bioactive compounds.

## Conclusions

Comparing with the Red Delicious apple, nine of the twelve fruits of the Cerrado studied; araticum, cagaita, cajuzinho, ingá, jenipapo, jurubeba, lobeira, magaba and tucum showed high levels of phenolic contents. The araticum and tucum are rich in flavonoids. The fruits cajuzinho, jatobá, jurubeba and tucum showed high content of anthocyanins. Cagaita, cajuzinho, lobeira, mangaba and tucum showed high levels of vitamin C. The high content of bioactive compounds found araticum, cagaita, cajuzinho, jurubeba, lobeira, magaba and tucum corresponded to the high antioxidant activity of these fruits. Daily consumption of at least seven of these Cerrado fruits studied, particularly, araticum, cagaita, lobeira, tucum may protect human tissues against oxidative stress, and thus, they may prevent chronic diseases and premature aging. Moreover, such fruits can provide a source of new bioactive compounds with functional properties beneficial to health, which should stimulate the pharmaceutical and food industries for the development of new products, promoting the sustainable development of regions with the characteristics of the Cerrado. New studies *in vivo* aimed at evaluating the antioxidant activity of these seven Cerrado fruits that showed high antioxidant potential are underway in our laboratory.
